# Impact of Birth Seasonality on Dynamics of Acute Immunizing Infections in Sub-Saharan Africa

**DOI:** 10.1371/journal.pone.0075806

**Published:** 2013-10-18

**Authors:** Audrey M. Dorélien, Sebastien Ballesteros, Bryan T. Grenfell

**Affiliations:** 1 Center for Social Epidemiology and Population Health, University of Michigan, Ann Arbor, Michigan, United States of America; 2 Department of Ecology and Evolutionary Biology, Princeton University, Princeton, New Jersey, United States of America; 3 Fogarty International Center, National Institutes of Health, Bethesda, Maryland, United States of America; University of Oxford, Viet Nam

## Abstract

We analyze the impact of birth seasonality (seasonal oscillations in the birth rate) on the dynamics of acute, immunizing childhood infectious diseases. Previous research has explored the effect of human birth seasonality on infectious disease dynamics using parameters appropriate for the developed world. We build on this work by including in our analysis an extended range of baseline birth rates and amplitudes, which correspond to developing world settings. Additionally, our analysis accounts for seasonal forcing both in births and contact rates. We focus in particular on the dynamics of measles. In the absence of seasonal transmission rates or stochastic forcing, for typical measles epidemiological parameters, birth seasonality induces either annual or biennial epidemics. Changes in the magnitude of the birth fluctuations (birth amplitude) can induce significant changes in the size of the epidemic peaks, but have little impact on timing of disease epidemics within the year. In contrast, changes to the birth seasonality phase (location of the peak in birth amplitude within the year) significantly influence the timing of the epidemics. In the presence of seasonality in contact rates, at relatively low birth rates (20 per 1000), birth amplitude has little impact on the dynamics but does have an impact on the magnitude and timing of the epidemics. However, as the mean birth rate increases, both birth amplitude and phase play an important role in driving the dynamics of the epidemic. There are stronger effects at higher birth rates.

## Introduction

The incidence of influenza and many other respiratory infections increases during cold winter months; cholera and malaria incidence increase during the rainy season; even the incidence of sexually transmitted disease such as gonorrhea increases during the summer months [1]–[4]. As these examples illustrate, seasonal fluctuations in the incidence of disease are common and have been documented for a range of diseases as early as circa 380 B.C. [5]. In recent decades, public health measures such as the World Health Organization’s (WHO) Expanded Program on Immunization and Supplementary Immunization Activities and the Measles Initiative have led to a reduction in incidence of many acute childhood immunizing (ACI) infections [6]. However many ACI diseases, such as measles, are still characterized by large episodic and seasonal outbreak, and remain significant killers of children in sub-Saharan Africa [7], [8]. A more detailed understanding of the disease drivers and the consequences for disease dynamics and control are needed.

A range of mechanisms with the potential to drive seasonal and multiannual fluctuations in the incidence of infectious diseases have been identified. They include factors that impact transmission, such as ability of the pathogen to survive outside a host, and seasonal changes in host behavior; factors that impact host susceptibility, such as seasonal changes in immune function; and factors that impact host birth rate, such as seasonal fluctuations in the birth rate [5], [9]–[11]. Despite some understanding of the proximate mechanisms that can create cyclical fluctuations, identifying the ultimate factors driving these processes (disease drivers) is difficult.

Most human disease ecologists have focused on identifying drivers leading to seasonal fluctuations in the transmission parameter, because of its large impact on infectious disease dynamics [11]. For strongly immunizing acute infections such as measles, seasonality in transmission (and stochastic forcing) can interact with the nonlinear epidemic clockwork to drive longer term epidemic oscillations [9], [11]–[13]. Transmission seasonality of measles has been found to be driven by aggregation of children during the school term in pre-vaccination England and Wales and by rural-urban migration in response to agricultural cycles in Niger and Cameroon [8], [14]–[18]. In contrast, relatively little research has been conducted on the epidemiological implications of seasonality in birth rates or host immune function [1], [11].

Almost all human populations exhibit seasonal variation in reproduction, which typically account of a large source of variation in birth rates [19]. Key features of birth seasonality are amplitude and phase. In this paper, amplitude refers to the maximum percent deviation from the mean, in this case the average birth rate, and the phase indicates the timing of the birth peak within the year (see Figure S1). Despite the ubiquitous presence of seasonal fluctuations in human birth rates, researchers have focused on the effects of slow changes in birth rates on disease dynamics. Researchers have clearly illustrated that changes in the baseline birth and vaccination rates can lead to dynamical transitions in periodicity of disease incidence and can influence the multiannual timing of an epidemic *in the presence* of transmission seasonality [20], [21]. Only one study thus far (summarized below) analyzed the impact of human birth seasonality as a driver of seasonal diseases [22].

This paper is an extension of the work of He and [22], who were the first to analyze the effects of human birth seasonality on infectious disease dynamics, to our knowledge. In their analysis of the impact of birth seasonality on infectious disease dynamics, He and Earn [22] focused on identifying whether increasing birth amplitude could create resonance (where amplitude of disease incidence is much larger than birth amplitude) and which combination of parameter ranges of 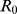 and duration of infectious period result in complex dynamics. As a result, they found that for most childhood infectious diseases, realistic levels of birth seasonality for developed countries do not induce resonance; furthermore, as birth amplitude increases, a wider range of 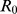 and infectious periods lead to complex dynamics. They also found that complex dynamics can occur in disease models with very short durations of infectious period (less than one day), even with low birth amplitude.

Other studies looking at the effect of intra-annual fluctuations in birth rates comes from studies of wildlife diseases [23]. However, unlike models of human diseases, these systems exhibit density-dependent host demography, short host life spans, and are often strictly constrained to a breeding season.

In this paper, we analyze the impact of seasonal fluctuations in human birth rates (birth seasonality) on measles disease incidence and dynamics. Not all infectious disease dynamics will be sensitive to seasonal forcing in the birth rates. Diseases of interest have the following properties–acute, immunizing infections, with a low mean age of infection [24]. Seasonality in birth rates will have the greatest impact on diseases where the majority of susceptibles are young [11]. We choose to model measles because it is a prototypical ACI infection.

**Figure 1:**
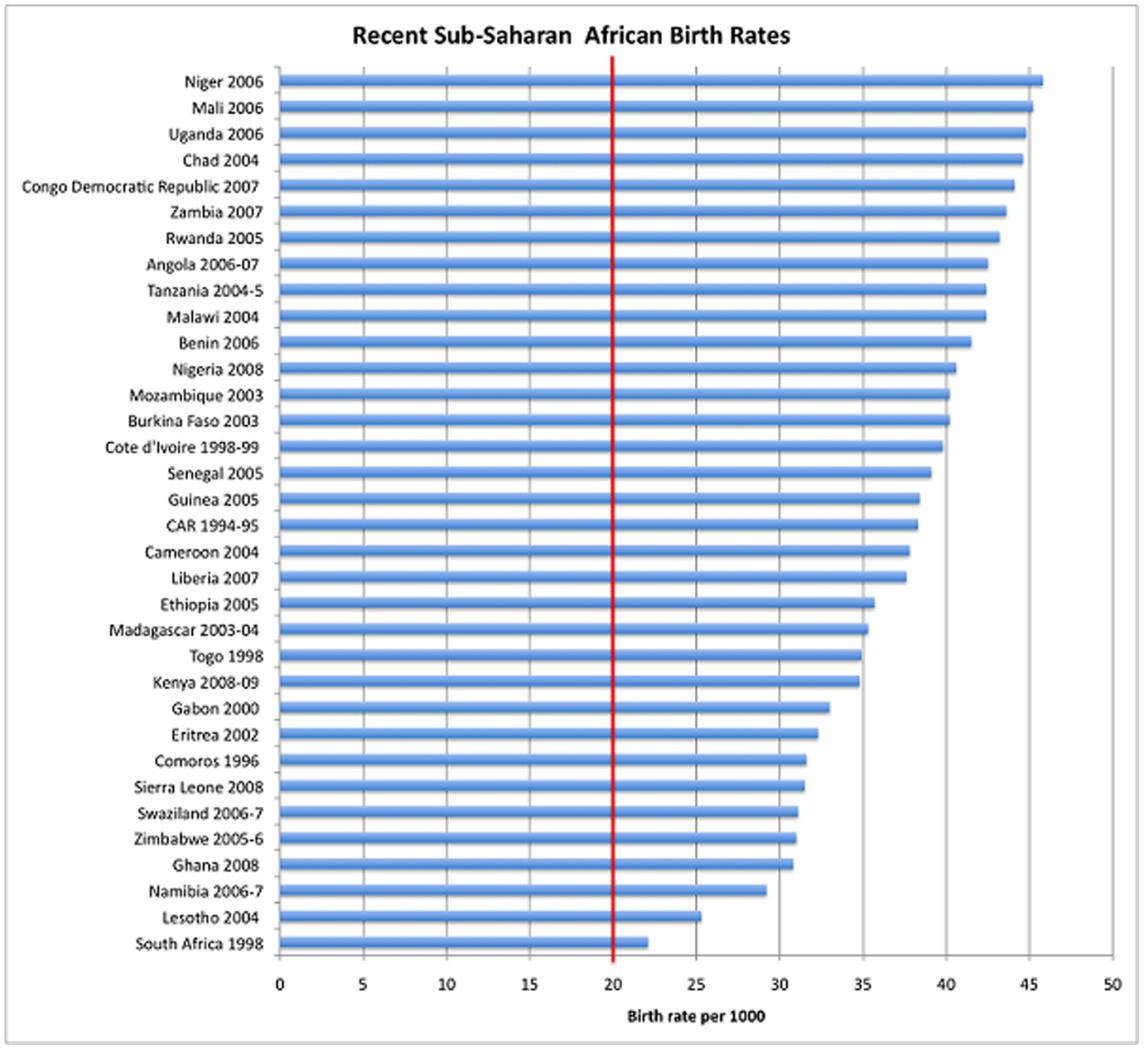
Recent Sub-Saharan African birth rates from the Demographic and Health Surveys. All of the SSA birth rates are above 20/1000, the baseline birth rate modeled by He and Earn [22].

**Figure 2:**
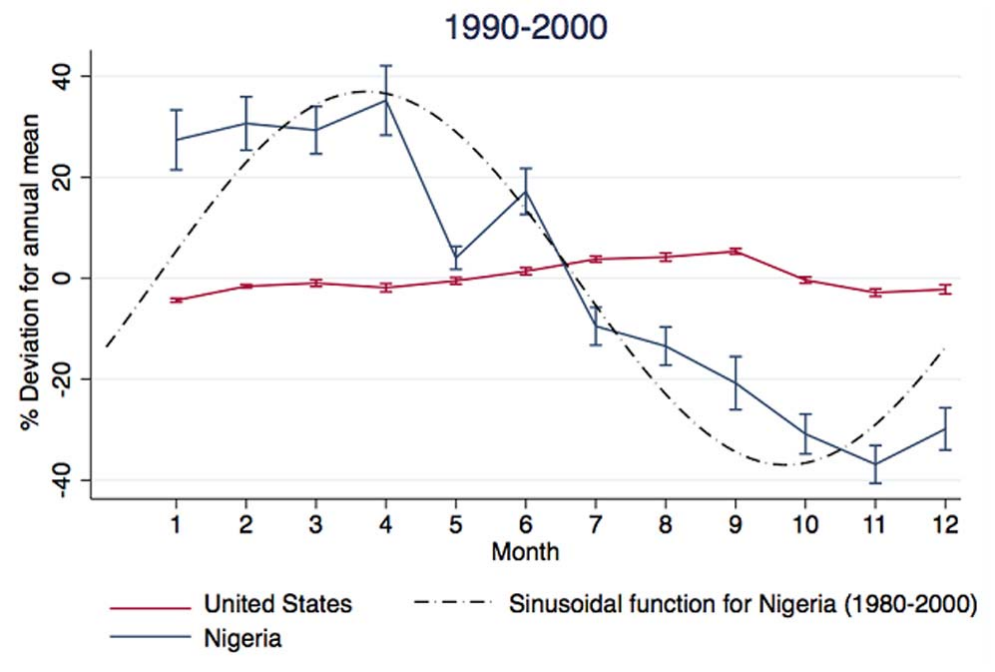
SSA birth rates are seasonal and exhibit some of the strongest birth fluctuations in modern times. Peak amplitude in Nigeria is above 30 percent while US peak amplitude is 6 percent. Vertical lines represent 95 percent confidence intervals. For many SSA countries, observed fluctuations in monthly birth amplitude can be represented by a sinusoidal function, as illustrated for Nigeria. Source: Annual summary of Monthly Vital Statistic Report, National Center for Health Statistics, United States; http://www/cdc.gov/nchs/births.htm Accessed 2010 June 21. Nigeria Demographic Health Surveys.

We focus on the effect of changes in birth amplitude given a wide range of baseline birth rates that corresponds to empirical data from sub-Saharan Africa, while He and Earn model a relatively low baseline birth rate (e.g., 20/1000) that corresponds to developed countries [22]. Birth seasonality is likely to have a strong impact on populations with high birth rates, and low vaccination levels. These conditions were present historically in developed countries, but still occur in many locations in sub-Saharan Africa (SSA) (Figure 1 illustrates the large birth rates typical of SSA). Furthermore, some of the largest birth amplitude levels in modern times are found in SSA. Figure 2 is a comparison of birth seasonality in the United States versus Nigeria. During the 1990s, amplitude in Nigeria was above 30 percent while amplitude in the US was approximately 6 percent. Therefore, in this paper we use demographic parameter ranges based on the much wider range in SSA.

Furthermore, we model the effect of birth seasonality on one disease, measles, so mean duration of the infectious period is constant and 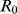 does not vary widely. We also extend our analysis in several ways. We analyze the impact of birth amplitude and phase on magnitude and timing of cyclical epidemics. We include both seasonal forcing in births (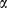) and transmission rates (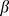).

Specifically, we try to answer the following question: what are the impacts of changing the parameters of the seasonal birth forcing function (e.g., baseline birth rate, amplitude, and phase; see Figure S1 for an illustration of the effects of changes in these parameters on birth rates) on infectious disease incidence and dynamics, in the absence and presence of seasonal transmission? It has been illustrated that changes in baseline birth rate and changes in baseline transmission rate lead to the same dynamical transitions Earn2000. Therefore, when we model the effects of changing baseline birth this has the same implications as changing baseline transmission rates. The intuition behind this is that the transmission rate has two components: contact rate and transmission probability. Increasing birth rate is like increasing the contact rate, which increases the transmission probability.

We identify plausible parameter ranges for both the baseline birth rates and birth amplitudes from analysis of sub-Saharan African (SSA) Demographic and Health Surveys (DHS). The DHS use nationally representative samples of women of reproductive age. The DHS contains retrospective birth histories, which have been widely used to estimate birth rates and recently seasonal birth amplitude. Although it had long been known that seasonal fluctuations in births are ubiquitous in human populations, there were very few estimates of birth seasonality in SSA, in part due to lack of vital registration data. Fortunately the DHS data can also be used to calculate birth seasonality. Following the method described by He and Earn [22, page 275], we obtain estimates of average monthly amplitude.

On average, SSA birth rates range from 22.1 in South Africa in 1998, to 51.7 per 1000 in Niger in 1998. We documented birth amplitude levels ranging from 5 to 65 percent at the national level. At the sub-national level, higher levels of birth amplitude have been documented, in SSA some in excess of 100 percent [25].

In addition to having an estimate for the strength of seasonality, we also need information on the shape of the seasonal forcing function [11], [20]. It has been shown that in the case of seasonality of transmission, the shape of the forcing function has large effects on the dynamics. As shown in Figure 2, we will assume a sinusoidal function for birth seasonality. For instance, we take the average monthly birth amplitude for Nigeria from 1980 to 2000, and using least squares estimation we are able to fit the data to the following function:
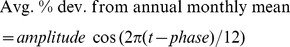
(1)


If the goal is to fit a disease model with birth seasonality, to observed data, we may choose to use actual monthly fluctuations in birth rates or birth amplitudes as parameters. Nevertheless, for the rest of this paper, a simple cosine function is used, as the shape of the seasonal recruitment function does not qualitatively affect whether bifurcations will take place [20].

## Methods

### 1 Measles Biology and the Seasonal SEIR Model

**Figure 3:**
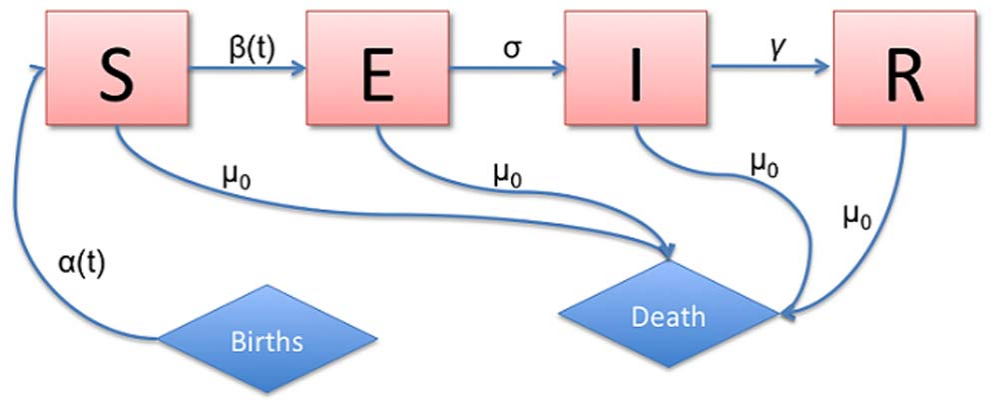
Transfer diagram for the SEIR model with seasonality in birth rate and transmission.

Measles is a highly infectious virus transmitted by aerosol particles [26]. It conforms well to assumptions of simple compartmental models with mean latent period of 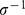 = 8 days and a mean infectious period of 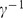 = 5 days. Furthermore, immunity is lifelong following both natural infection and seroconversion after administration of the live attenuated vaccine against the disease [26]. In this paper we use a compartmental model, illustrated in Figure 3, that divides the population into susceptible (S), exposed but not yet infectious (E), infectious (I), and recovered (R) groups, referred to as SEIR, to simulate the propagation of an immunizing non-fatal acute disease in a stable and well-mixed population with frequency-dependent transmission. For simplicity we do not use age-structured models which are common for childhood infectious diseases. Our model differs from standard SEIR models in that both birth and transmission rates can be seasonally forced (i.e., can vary overtime in a cyclical manner).

The rates of change in the compartments is defined by the following differential equations:
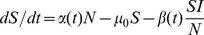
(2a)

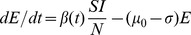
(2b)

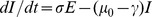
(2c)

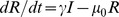
(2d)


Here, the seasonal forcing functions–birth seasonality (which in this case refers to births of susceptibles post waning maternal immunity) 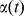 and frequency dependent transmission seasonality 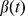 are given by:
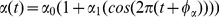
(3)

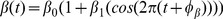
(4)


The *per capita* natural death rate (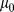) is equal to the baseline *per capita* birth rate (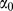). The birth rate 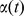 and transmission rate 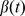 vary seasonally around their respective baseline values (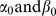), with an amplitude equal to 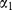 and 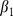. The parameter 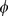 (phase), with values between 0 and 1, indicates the location of the peaks within the year. The mean duration of the latent period and infectious period are 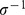 and 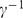, respectively.

### 2 Analysis

We solve the model by numerical integration using the Runge-Kutta-Fehlberg (4, 5) integration method from the GNU Scientific library. Starting with a population where the proportion of susceptible individuals are six percent of the population, exposed and infectious individuals are each 0.1 percent of the population, and recovered individuals represent 93.8 percent of the population and parameter values (e.g., 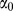
…), the model is run for at least a 1000 years so that the dynamics have reached their attractor. “It is possible to observe qualitatively different dynamics (or multiple attractors) for the same combination of parameter values, depending on initial conditions” [27]. Therefore, in regions with coexisting attractors, we may need to sample a large set of initial starting conditions to see if there are any qualitative differences. We then view and analyze the results using plots, bifurcation diagrams, and heat maps of bifurcation diagram results (a.k.a., two-parameter bifurcation diagrams) and incidence. See below for details of these methods.

We calculate the periodicity of the epidemic cycle in two ways. The first method involves calculating the period of the attractor. Discarding the first 1000 years of the simulation, we identify the peaks, and then calculate the time it takes for the trajectory to reach the peak value a second time. If that peak value occurs the following year, we can deduce that the pattern is annual, if the peak appears two years later, it is biennial, and so on. We will call this measure the period of the attractor (blue circles in Figure S2).

Following the formula in Keeling and Rohani [27], the dynamics are of period 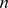 if:
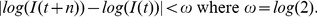
(5)


We also calculate the dominant period of the epidemic cycle using Fourier spectra (background of Figure S2 the peaks are in red). This method informs us on the major components of the periodicity. For instance, we may have a strictly four year cycle but the dominant period may be two years; which may mean that we have a high-low-high-low pattern, where the two peaks are not of the same magnitude. A major weakness of this strategy is that even when a cycle is multiennial or chaotic, the annual signature may dominate (due to the seasonal forcer). Therefore we also calculate the Lyapunov exponents to determine whether the dynamics are indeed chaotic [28]. Lyapunov exponents are the exponential rates at which two nearby orbits diverge or converge from each, therefore they are crude measures of chaotic behavior. If the main Lyapunov exponent is positive (nearby points diverge from each other), then the dynamics are said to be chaotic; when the system bifurcates the Lyapunov exponent equals zero.

As mentioned above, the model is sensitive to starting conditions, so the bifurcation diagrams are run with a fixed set of initial starting conditions, in addition to extrapolated starting conditions. Nevertheless, because of the influence of initial starting conditions, a large sample of bifurcation diagrams is needed to model the range of possible behavior.

## Results

### 1 Birth Seasonality in the Absence of Seasonal Transmission

#### Impact on dynamics

**Figure 4:**
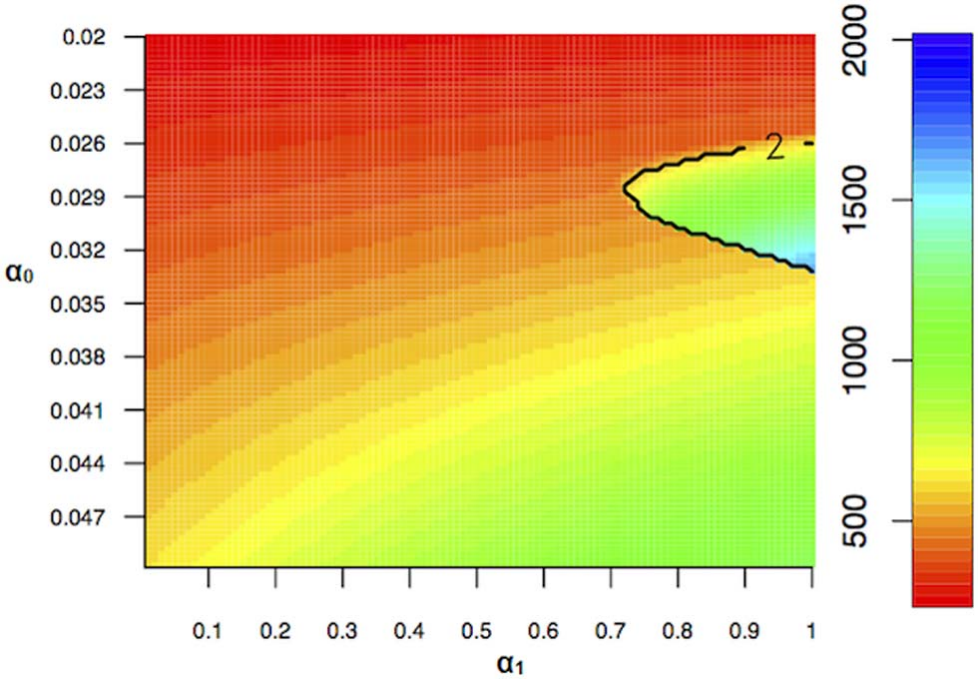
Heat map illustrating how incidence of epidemics change with changing birth rate and amplitude. The contour plot illustrates the transition from annual to biennial epidemics. The timing of the epidemic did not change significantly with changing birth rate (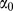) and amplitude (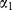). (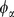 = 0, 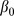 = 1000, 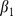 = 0).

As expected, the seasonal fluctuations in the birth rate can lead to oscillations in disease incidence. In the absence of seasonal transmission rates, seasonal birth rates can lead to annual and biennial dynamics, as shown in Figure 4. Which type of dynamic we observe depends on the baseline birth rate and transmission rate as well as the birth amplitude.

Low and high levels of baseline birth rate and transmission rates (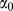 and 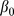) lead to annual epidemics regardless of the magnitude of birth amplitude. This is a similar finding to what is found in density dependent models [27]. At intermediate ranges of baseline birth rate (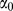) and transmission rates, high levels of birth amplitude can lead to biennial dynamics (Figure 4 and Figure S3). If transmission rates are very large, than lower levels of baseline birth rates are needed for birth amplitude to induce biennial dynamics.

**Figure 5:**
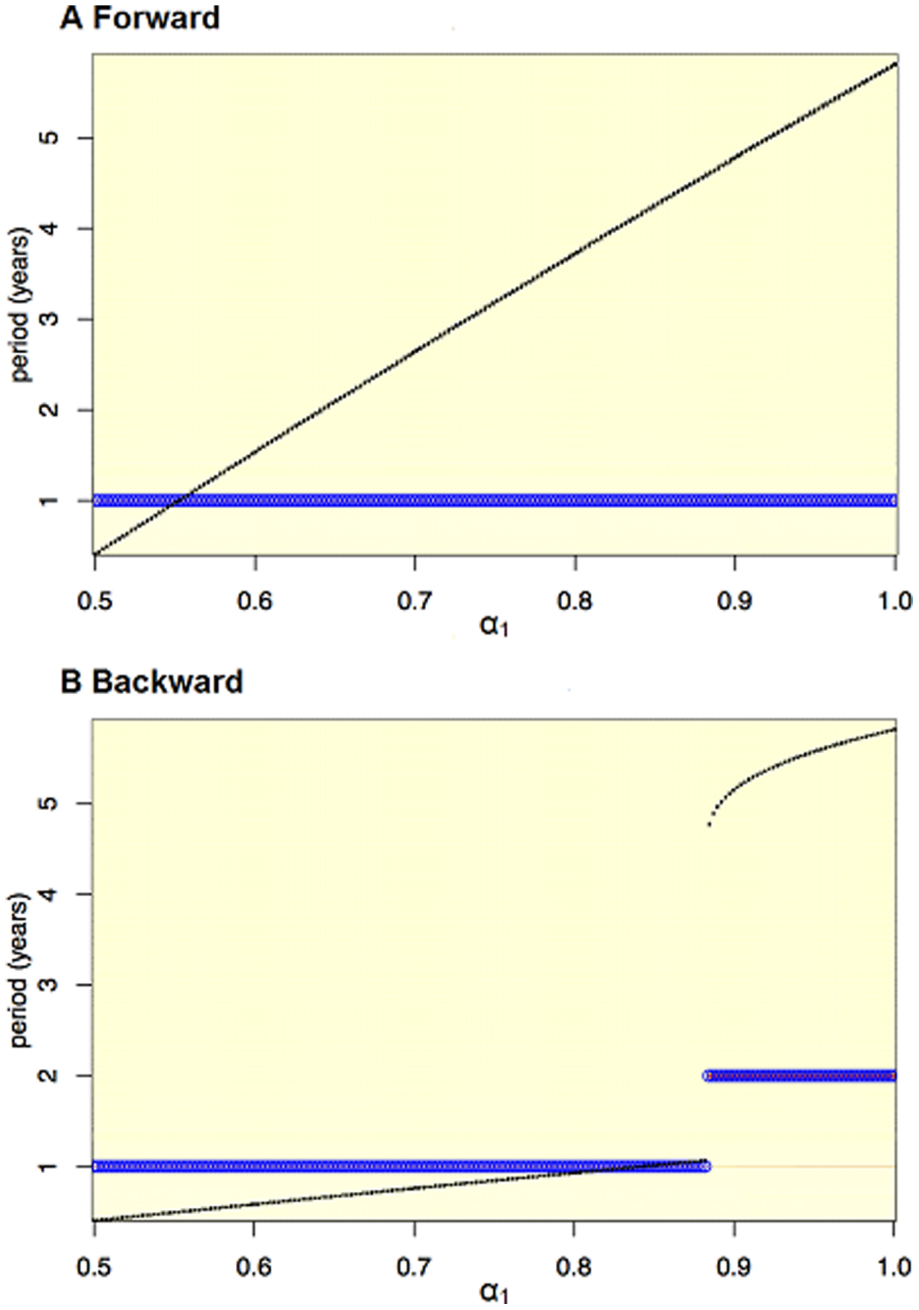
Bifurcation diagrams showing the impact of varying birth amplitude (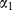) on the periodicity of the epidemics. Simulations use extrapolated initial conditions (the numbers of susceptibles, exposed, infectives, and recovered at the end of one simulation are used to start the next simulation [27]). In Panel A the first simulation is at 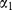 = 0 (left hand of the x axis), then 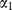 is increased in the subsequent simulations; to further sample the bifurcation structure, Panel B reverses this order, starting at 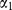 = 1. Black points represent the relative size of the incidence peaks, blue circles represent the period of the attractor, while the background is a heat map of the power spectral densities where the color red signifies higher power. In Panel B, at high levels of 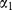, there are both annual and biennial components present but the power is stronger for the biennial component. (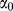 = 35/1000, 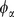 = 0, 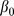 = 1000, 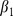 = 0).

For high parameter values of birth amplitude (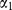) and some values of 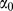, the dynamics depend in part on the initial starting conditions. For instance, in Figure 5, the parameters are the same, however Panel A is constructed using extrapolated initial conditions (the numbers of susceptibles, exposed, infectives, and recovered at the end of one simulation are used to start the next simulation [27]) starting at 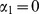, while Panel B is constructed using starting conditions 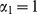. In the former, the resulting epidemics are always annual, while in the latter for large values of birth amplitude, the dynamics are biennial. These results indicate that these are regions with coexisting annual and biennial attractors. He and Earn also identified regions with coexisting attractors [22].

#### Impact on disease incidence

As predicted by He and Earn [22], we do not find any resonance (the amplitude of disease incidence is not greater than the birth amplitude). In the regime of annual attractors, the size of the epidemic increases with increasing birth rate and birth amplitude. At higher birth rates (birth rates greater than 30 per 1000) the influence of changing birth amplitude is magnified (Figure 4).

#### Impact on timing of disease peak

Pitzer et al. [21] illustrated that spatiotemporal variation in birth rates in the presence of transmission seasonality explained the timing of rotavirus epidemics in the United States. Therefore we are interested in the influence of birth rate and birth amplitude on timing of disease epidemics in the absence of transmission seasonality. As the baseline birth rate increases, the epidemic occurs slightly earlier in the year, but as the amplitude increases, the epidemic occurs slightly later in the year. As expected, the phase has a stronger influence on the timing of the peaks compared to amplitude (not shown).

### 2 Birth Seasonality in Presence of Seasonal Transmission

**Figure 6:**
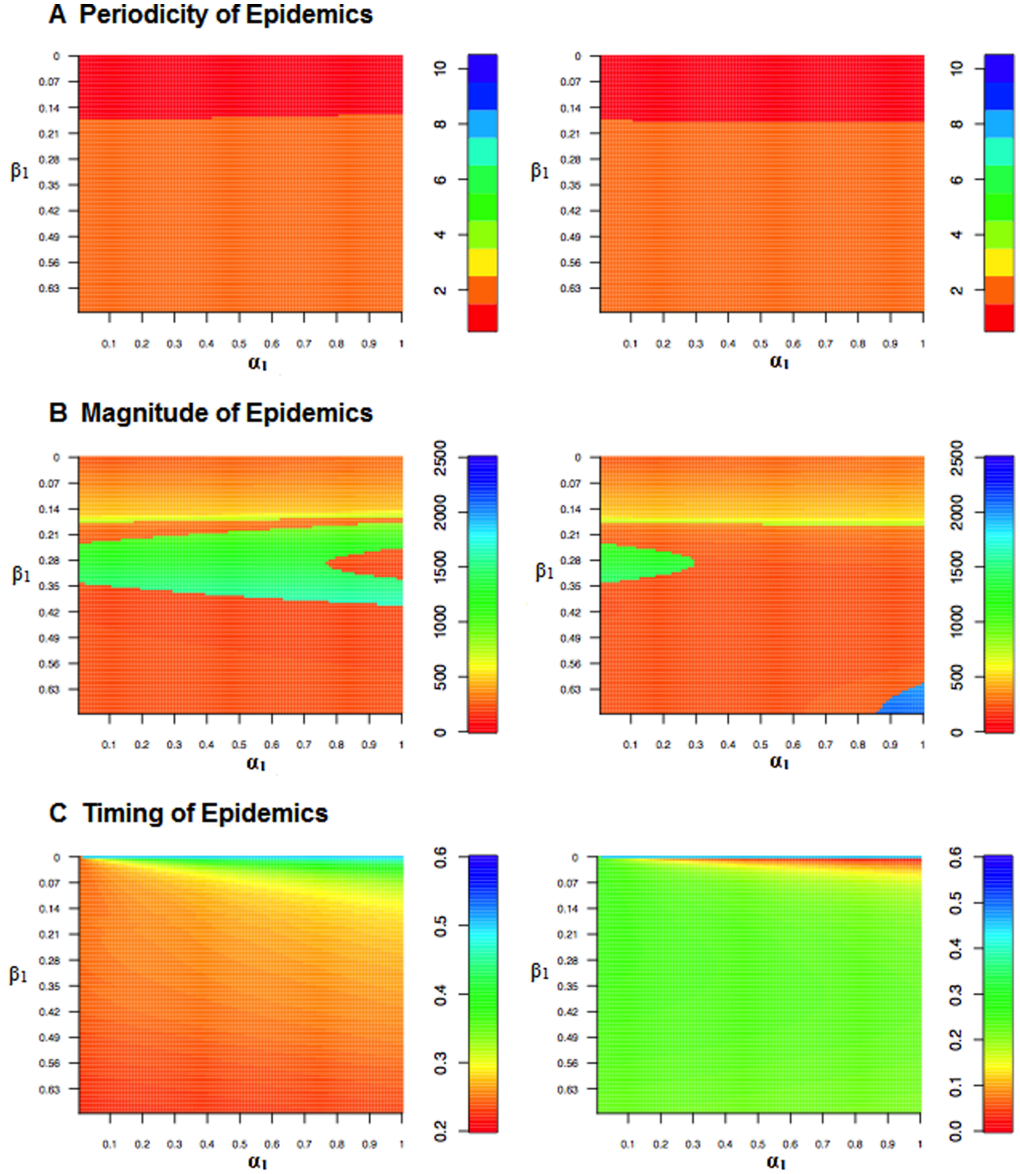
Heat maps illustrating how periodicity, incidence, and timing of epidemics change with varying levels of transmission (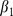) and birth (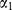) amplitudes. In the figures on the left, the two seasonal forcers have the same phase, in the figures on the right, the phases are out of synch. (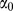 = 20/1000, 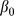 = 1000).

According to He and Earn [22], “even with large phase differences between the two seasonal forcing functions, inclusion of birth seasonality will not have substantial effects on asymptotic dynamics for parameter ranges that correspond to known infectious diseases.” That statement is correct when the baseline birth rate is low (e.g., 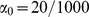). In Figure 6, we run a model with both birth and transmission seasonality and analyze the impact of varying both the birth and transmission amplitudes on the periodicity of the disease dynamics (Panel A), and the magnitude (Panel B) and timing of the epidemics (Panel C). It is clear that at low birth rates, it is the seasonality in the transmission parameter that is having the largest influence on the periodicity of the epidemics, changing birth amplitude has very little effect on determining whether the epidemics are annual or biennial (Figure 6a). Nevertheless, the interaction between birth and transmission amplitude, as well as the phase, does influence the magnitude and timing of the epidemics.

Once the periodicity of the epidemic changes from annual to biennial epidemics, large changes in the magnitude of the epidemic peak are not concomitant with changes in the periodicity; both changes in the transmission and birth amplitudes can lead to large changes in the magnitude of the epidemic peaks (Figure 6b). At very low levels of transmission seasonality, increases in the birth amplitude shifts the timing of the epidemic peak towards the end of the year when transmission and birth seasonality are in-phase, but as the transmission amplitude increases the effects of increases in birth amplitude on the timing of the epidemic peak diminishes. When seasonal fluctuations in birth and transmission rates are anti-phase, increases in the birth amplitude shift the timing of the epidemic peaks towards the beginning of the year; however the timing of the epidemic peak does not change significantly once transmission amplitude rises above 10 percent (Figure 6c).

**Figure 7:**
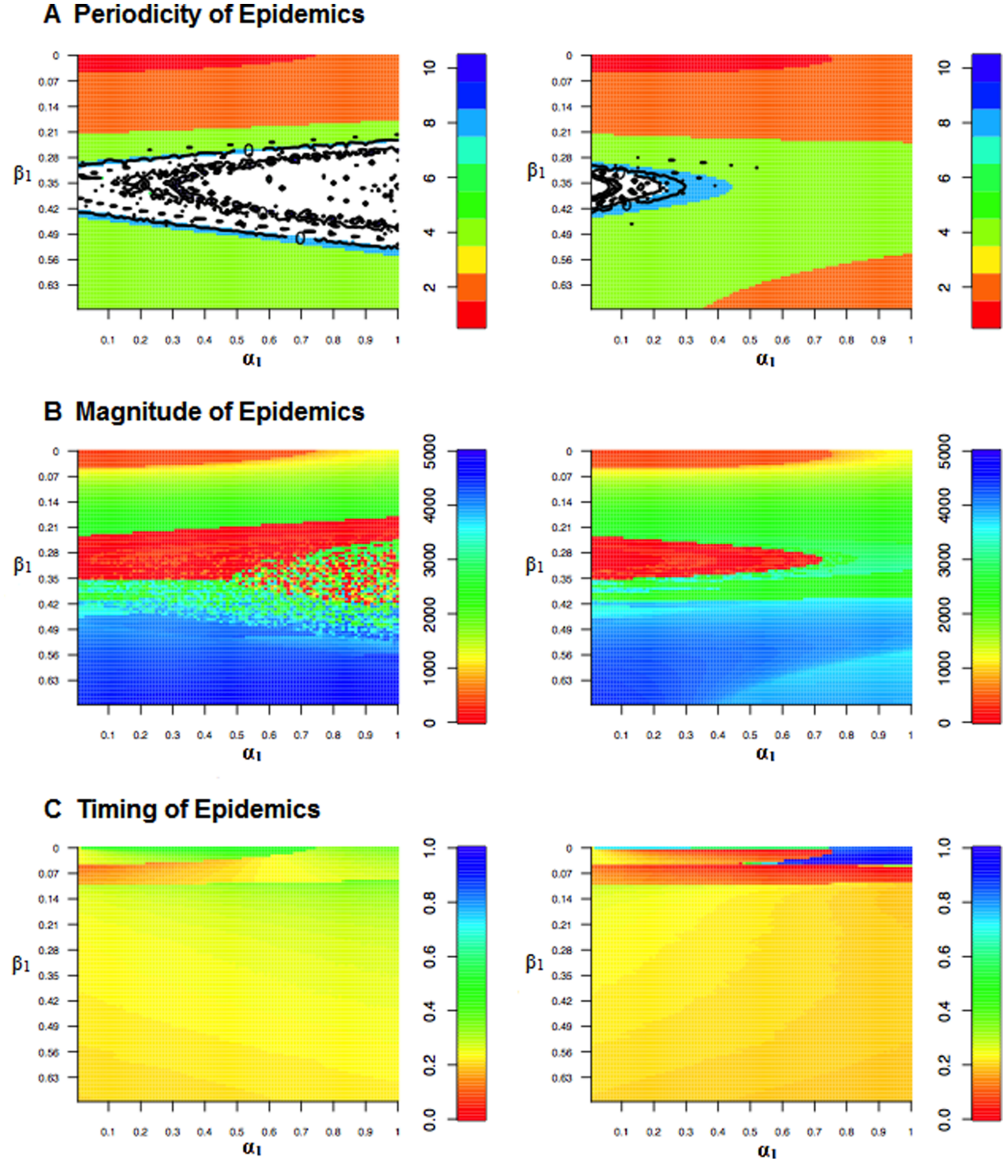
Heat maps illustrating how periodicity, incidence, and timing of epidemics change with varying levels of transmission (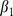) and birth (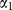) amplitudes. In the figures on the left, the two seasonal forcers have the same phase, in the figures on the right, the phases are out of synch. In Panel A, values in white are chaotic; the contour plots indicate where the main Lyapunov exponent crosses zero. (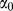 = 30/1000, 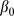 = 1000).

**Figure 8:**
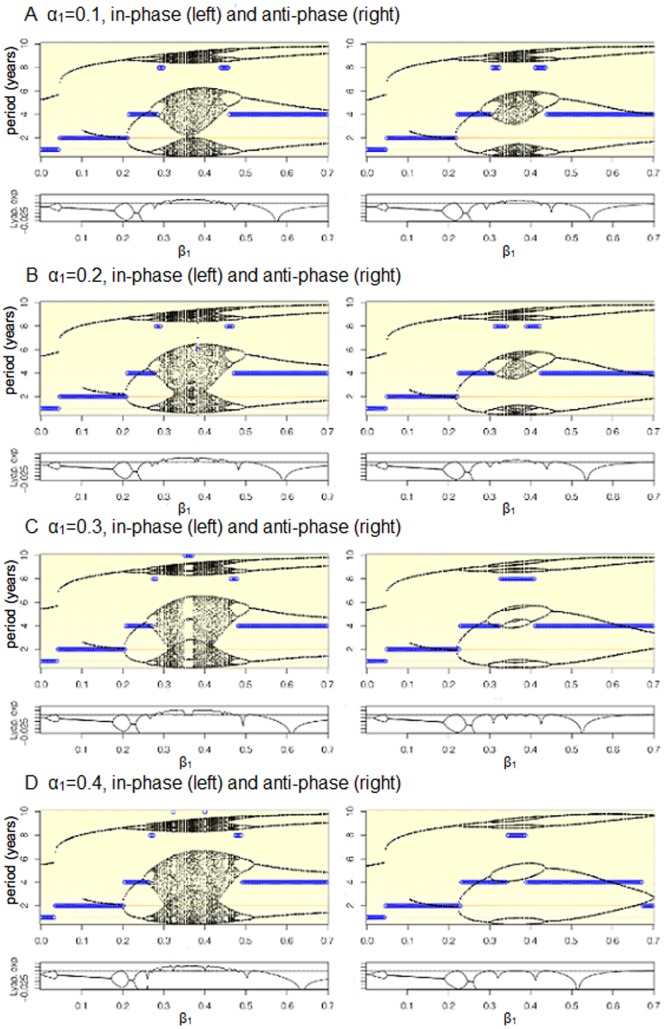
One-dimensional bifurcations of the effect of varying 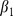 values given different baseline values of 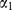 (0.1, 0.2, 0.3, 0.4) and 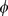 (in phase, anti-phase). Black points represent the relative size of the epidemic peaks, blue circles represent the period of the attractor, while the background is a heat map of the the power spectral densities where the color red signifies higher power. In all of the figures, the presence of an annual and biennial component is always present even though, for instance, an attractor may have a period of four years. The bottom panel in each of the figures shows the main Lyapunov exponent, when the system bifurcates the Lyapunov exponent equals zero (touches the horizontal line), when the Lyapunov exponent is greater than zero the dynamics are said to be chaotic. (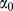 = 30/1000, 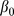 = 1000 in all panels).

**Figure 9:**
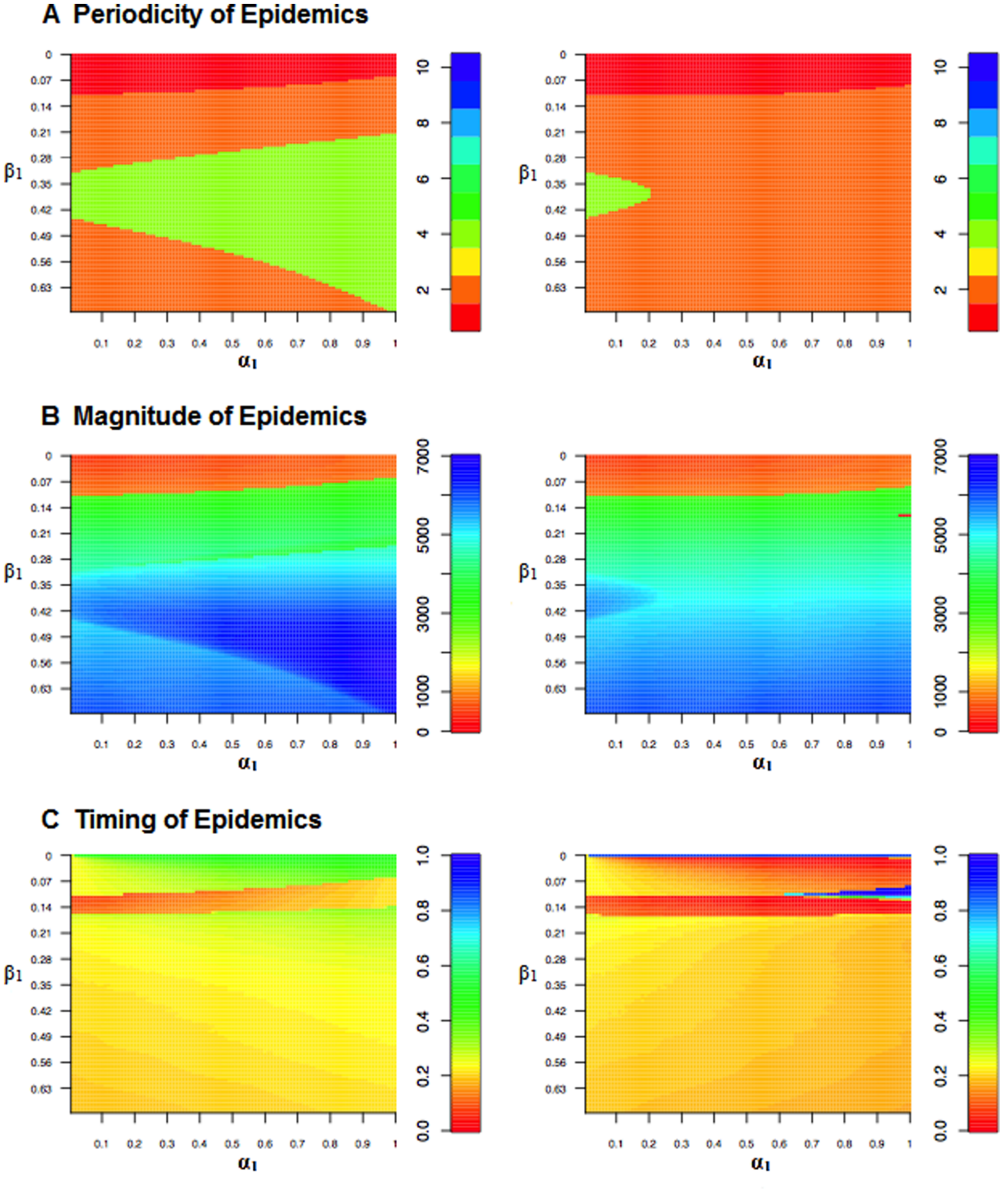
Heat maps illustrating how periodicity, incidence, and timing of epidemics change with varying levels of transmission (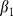) and birth (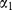) amplitudes. In the figures on the left, the two seasonal forcers have the same phase, in the figures on the right, the phases are out of synch. (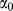 = 40/1000, 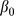 = 1000).

At higher birth rates, as in SSA, phase differences between the two forcing functions do have a substantial effect on the dynamics (Figures 7–9). Specifically, when birth and transmission seasonality are in-phase, increasing birth amplitude exacerbates the tendency for chaotic dynamics; when in anti-phase, increasing birth amplitude stabilizes the dynamics (Figures 7a, 8, and 9a). The latter is illustrated in Figure 7a by looking at the rows that corresponds to 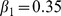 when birth and transmission seasonality are in-phase, for almost all values of 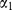, the resulting dynamics are chaotic, but when birth and transmission seasonality are anti-phase, increasing 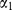 changes the dynamics from chaotic, to an eight year cycle, then to a four year cycle. Another way to understand the effect of the differences in-phase is to look at Figure 8, which contains one dimensional bifurcation plots of the effect of varying 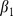 given different baseline values of 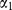, and plots of the main Lyapunov exponent. We find that when the two forcing functions are in anti-phase the region with chaotic dynamics is smaller than when they are in phase, this is especially true for large values of birth amplitude (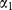). When there are no differences between the phase, increasing birth amplitude expands the range of 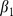 values that result in chaotic dynamics; when the phase are asynchronous, the range of 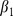 values that result in chaotic dynamics decreases as birth amplitude increases.

At high baseline birth rates, the interactions between the amplitude and phase of the seasonal birth and transmission rates also influence the magnitude and timing of the epidemic peaks (Figures 7b–c and 9b–c). Changes in the magnitude of the epidemic peak correspond to changes in the periodicity of the epidemic (Figures 7b and 9b); this was not the case at low baseline birth rates (Figure 6b). Furthermore, as the baseline birth rate increases, the synchrony between the changes in timing and periodicity also increases.

When transmission and birth seasonality are in-phase, above a threshold level of transmission amplitude (which rises with baseline birth rates), increasing transmission amplitude above the threshold shifts the epidemic towards the beginning of the year while increasing birth amplitude shifts the timing of the epidemic peaks towards the end of the year. When birth and transmission seasonality are out of phase, above the aforementioned threshold, increasing both transmission and birth amplitudes shift the timing of the epidemic peak to the beginning of the year (Figures 7c and 9c). Below the threshold, the effects of changing both birth and transmission amplitude on the timing of the epidemic peak are more varied but appear to be in part influenced by periodicity of the epidemics.

## Discussion and Conclusion

In this paper we extend previous work to show that at high birth rates, amplitude can be important, especially coupled to seasonality in transmission. We also highlight an important source of data, the Demographic and Health Surveys, for estimating seasonal birth patterns for many SSA countries. Having an estimate of the birth seasonality function, allows us to separate seasonal forcing in births from other seasonal forcers such as those in transmission.

Models of acute immunizing childhood infections in SSA should take into account birth seasonality. The presence of birth seasonality alone can lead to spatial and temporal variation in the periodicity, magnitude, and timing of epidemics in SSA. The interaction between baseline birth rate and birth amplitude are important. Biennial epidemics occur only at high levels of birth amplitude and intermediate levels of baseline birth rates. In the regime of annual epidemics, as baseline birth rate increases the effect of increasing birth amplitude on peak incidence also increased.

We also explored the interactions between transmission and birth seasonality. As He and Earn [22] stated, at low levels of birth rate, there are no strong interactions between seasonality in birth and contact rates. However, we show that at the high birth rate levels found in many SSA countries, birth amplitude and phase significantly impact disease dynamics in the presences of seasonality in contact rates. Specifically, at higher birth rates, when birth and transmission seasonality are in-phase, increasing birth amplitude tends to exacerbate chaotic dynamics; when they are out of phase, increasing birth amplitude tends to stabilize the dynamics.

There are some limitations to this study. We did not use an age-structured model; however, the SEIR model without age structure still captures the dynamical essence of the system [20]. We focused on the effects of changing the baseline birth rate and its degree of seasonality, however in SSA, under-five mortality is high and may also oscillate seasonally. In the model, we set baseline birth and death rates equal, therefore when birth rate were high so was the death rate. Furthermore, in models with frequency dependent transmission increases in death rates have a smaller effect than in density-dependent transmission where changes in the population size play an important role in transmission. Nevertheless, a high under-age five death rate could act to dampen the effects of seasonality in births if deaths are random and aseasonal. Finally, we presented the results of the analysis based on deterministic models, however in File S1, we also conducted the analysis using a model with demographic stochasticity. The presence of stochasticity in an SEIR model with birth seasonality tends to lead to biennial disease dynamics. However, at high birth rates and extremely high birth amplitude the dynamics began to resemble those of the deterministic model.

### Future Work

Candidate SSA countries where birth seasonality may play an important role include Sierra Leone, Guinea, Democratic Republic of Congo, and Nigeria. These countries have strong birth seasonality (maximum amplitude between 30 to 65 percent), low proportions of fully immunized population (20 to 40 percent), and high birth rates (30/100 to 44/1000)] [29]. We will try to obtain monthly measles incidence for these countries in order to test the predictive capabilities of a statistically-fitted SEIR model with seasonal birth and/or seasonality in contact rates. Alternatively we will also try to use historical data from developed countries to test our model. For instance, weekly ACI disease incidence is available for pre-vaccination Copenhagen [30], but preliminary analysis of Copenhagen birth data revealed low birth seasonality (five percent from 1900–1937).

Birth amplitude tends to decline over time, as a result of increased development [31]–[33]. In SSA higher socio-economic status, improved housing and other indicators of wealth are associated with lower birth seasonality; these factors appear to shield populations from the influence of birth seasonality drivers. As the demographic transition and birth amplitude declines in SSA, we expect birth seasonality to have less of a role in influencing acute childhood immunizing (ACI) disease dynamics.

## Supporting Information

Figure S1
**Effects of changing the parameters (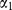, 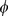, 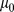) in the birth seasonality forcing function.**
(TIFF)

Figure S2
**This bifurcation diagram illustrates the impact of changing magnitude of 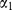 on the size of the relative size of the epidemic peaks (black dots), the dominant period (yellow background, peaks are in red), and the period of the attractor (blue circles).** Two trajectories are for 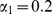 and 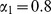. No chaotic dynamics appear. We have annual or biennial epidemics. After the bifurcation, the dominant period is two years, but there are still annual epidemics. (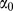 = 30/1000, 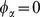, 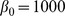, 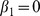).(TIFF)

Figure S3
**Bifurcation diagram at the Poincaré section showing the impact of varying birth amplitude (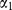) for different values of baseline birth rates (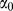).** Single circle indicates that the period is annual, two circles indicate that the period is biennial. At low birth rates (20/1000) increasing the amplitude leads to larger annual epidemics. At intermediate birth rates (30/1000) increasing amplitude first leads to larger annual epidemics but at amplitudes greater than 65 percent increasing amplitudes lead to biennial epidemics with increasing peak sizes. At very high birth rates (40/1000) changes in amplitude lead to increasingly large annual epidemics. (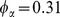, 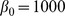, 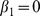).(TIFF)

Figure S4 **Spectral analysis and relative size of peak incidence in model with demographic stochasticity.** The peaks (black dots) are not of the same magnitude from year to year but a general pattern emerges as we increase amplitude and birth rates. We use heat colors to indicate the power spectral density, therefore the highest peaks are in red.
(TIFF)

File S1 **Analysis of a Stochastic SEIR model with seasonality in the birth rate.** The periodicity and relative changes in peak incidence are plotted for varying levels of birth amplitude and baseline birth rates in Figure S4.
(PDF)
