# Supplementary figures and images for: Impact of Birth Seasonality on Dynamics of Acute Immunizing Infections in Sub-Saharan Africa
Source: PLoS One. 2013 Oct 18;8(10):e75806. doi: 10.1371/journal.pone.0075806 (PMC3799982; doi:10.1371/journal.pone.0075806)

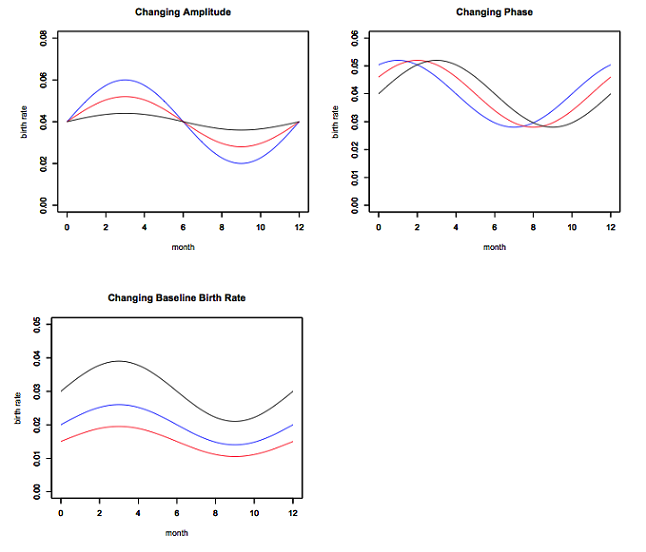

Supplement: Figure S1 — Effects of changing the parameters (, , ) in the birth seasonality forcing function. (TIFF) [file pone.0075806.s001.tiff]

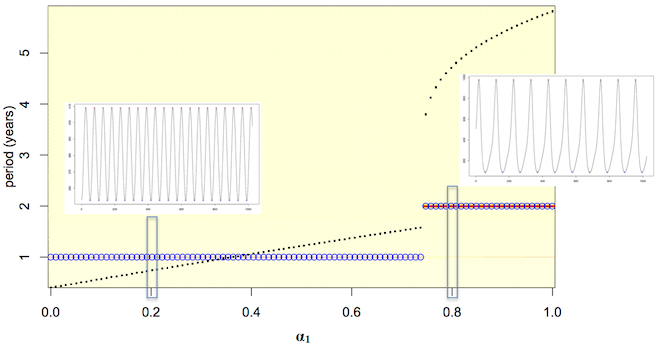

Supplement: Figure S2 — This bifurcation diagram illustrates the impact of changing magnitude of on the size of the relative size of the epidemic peaks (black dots), the dominant period (yellow background, peaks are in red), and the period of the attractor (blue circles). Two trajectories are for and . No chaotic dynamics appear. We have annual or biennial epidemics. After the bifurcation, the dominant period is two years, but there are still annual epidemics. ( = 30/1000, , , ). (TIFF) [file pone.0075806.s002.tiff]

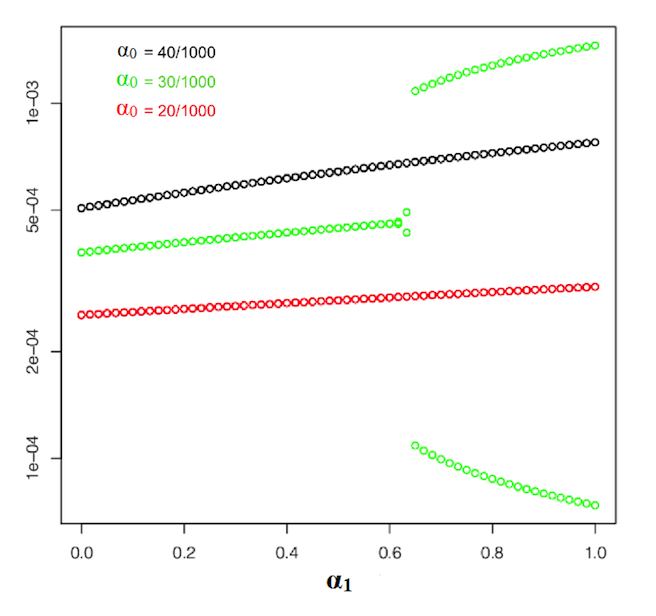

Supplement: Figure S3 — Bifurcation diagram at the Poincaré section showing the impact of varying birth amplitude () for different values of baseline birth rates (). Single circle indicates that the period is annual, two circles indicate that the period is biennial. At low birth rates (20/1000) increasing the amplitude leads to larger annual epidemics. At intermediate birth rates (30/1000) increasing amplitude first leads to larger annual epidemics but at amplitudes greater than 65 percent increasing amplitudes lead to biennial epidemics with increasing peak sizes. At very high birth rates (40/1000) changes in amplitude lead to increasingly large annual epidemics. (, , ). (TIFF) [file pone.0075806.s003.tiff]

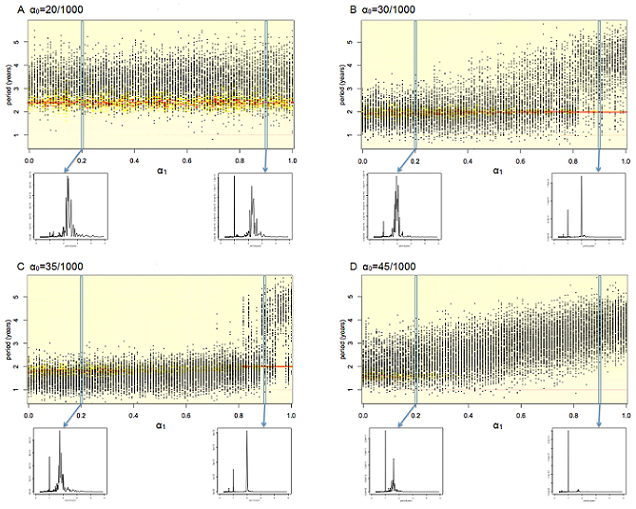

Supplement: Figure S4 — Spectral analysis and relative size of peak incidence in model with demographic stochasticity. The peaks (black dots) are not of the same magnitude from year to year but a general pattern emerges as we increase amplitude and birth rates. We use heat colors to indicate the power spectral density, therefore the highest peaks are in red. (TIFF) [file pone.0075806.s004.tiff]
