# Supplementary material for: Impact of Birth Seasonality on Dynamics of Acute Immunizing Infections in Sub-Saharan Africa
Source: PLoS One. 2013 Oct 18;8(10):e75806. doi: 10.1371/journal.pone.0075806 (PMC3799982; doi:10.1371/journal.pone.0075806)
Supplement: File S1 — Analysis of a Stochastic SEIR model with seasonality in the birth rate. The periodicity and relative changes in peak incidence are plotted for varying levels of birth amplitude and baseline birth rates in Figure S4. (PDF) [file pone.0075806.s005.pdf]

File S1. Stochastic SEIR Model

In this supplement we introduce an SEIR model with demographic stochasticity ("fluctuations in population processes that arise from the random nature of events at the level of the individual"[2, page 201]) . In the stochastic model we do not assume a continuous state space (i.e., there are no fractional individuals as in the deterministic model). The stochastic SEIR model is simulated using the tau-leap method for a population of one million with a small import parameter τ [1–3]. We are interested in learning whether there are qualitative differences in the impact of birth seasonality on disease dynamics. The motivation comes from the fact that even in the absence of birth seasonality, stochasticity can induce oscillations in incidence [3, 4]. Furthermore, He and Earn [5] suggested that transient dynamics may be sustained by demographic stochasticity. Specifically, periodicity of the epidemic cycles may deviate from the periodicity "predicted" by the deterministic model [3]. Therefore, we would like to identify for which parameter values of the seasonal birth functions, the stochastic model resembles the deterministic model.

The simulations reveal that there are qualitatively different dynamics in a model with demographic stochasticity. At low birth rates, the dominant period of the epidemic cycles is not annual and appears to be similar to that of the damped oscillator in the deterministic model (Figure S4). However at high baseline birth rates and amplitude the dynamics of the stochastic and deterministic model are similar– the periodicity of the dynamics are annual (Figure S4 panel d). At intermediate birth rate levels for low levels of birth amplitude the dominant periods is roughly two years but as the amplitude increase the dominant period becomes more of a sharp two year cycle (Figure S4 panel b and c). The epidemic incidence does appear to increase with increasing amplitude and as in the deterministic model, the effect is magnified at higher base line birth rates.

References

1. Drake JM (2010) Simulating epidemics in R. Notes from Ecology and Evolution of Infectious Disease Workshop.

2. Keeling MJ, Rohani P (2008) Modeling Infectious Diseases in Humans and Animals. Princeton University Press.

3. Keeling M, Ross J (2008) On methods for studying stochastic disease dynamics. Journal of The Royal Society Interface 5: 171-181.

4. Bartlett MS (1956) Deterministic and stochastic models for recurrent epidemics. In: Proc. Third Berkeley Symp., Mathematics, Statistics and Probability. University of California Press, volume 4, pp. 81-109.

5. He D, Earn D (2007) Epidemiological effects of seasonal oscillations in birth rates. Theoretical Population Biology 72: 274-291.
